# Decoupling the Influence of Poly(3,4‐Ethylenedioxythiophene)‐Collagen Composite Characteristics on Cell Stemness

**DOI:** 10.1002/advs.202305562

**Published:** 2024-02-13

**Authors:** Rebecca L. Keate, Joshua Tropp, Ruiheng Wu, Anthony J. Petty, Guillermo A. Ameer, Jonathan Rivnay

**Affiliations:** ^1^ Department of Biomedical Engineering Northwestern University Evanston IL 60208 USA; ^2^ Center for Advanced Regenerative Engineering Northwestern University Evanston IL 60208 USA; ^3^ Simpson Querrey Institute Northwestern University Chicago IL 60611 USA; ^4^ Department of Surgery Feinberg School of Medicine Northwestern University Chicago IL 60611 USA; ^5^ Chemistry of Life Processes Institute Northwestern University Chicago IL 60208 USA; ^6^ International Institute for Nanotechnology Northwestern University Chicago IL 60208 USA

**Keywords:** collagen, conductive polymers, dopant, pluripotency, stemness

## Abstract

Conductive polymers (CPs) are widely studied for their ability to influence a myriad of tissue systems. While their mixed ionic/electronic conductivity is commonly considered the primary driver of these benefits, the mechanisms by which CPs influence cell fate remain unclear. In this study, CP‐biomaterial interactions are investigated using collagen, due to its widespread prevalence throughout the body and in tissue engineering constructs. Collagen is functionalized with both electrostatically and covalently bound derivatives of the CP poly(3,4‐ethylenedioxythiophene) (PEDOT) doped via backbone‐tethered sulfonate groups, which enable high solubility and loading to the collagen biomatrix. Intrinsically doped scaffolds are compared to those incorporated with a commercially available PEDOT formulation, which is complexed with polyanionic polystyrene sulfonate (PSS). Low loadings of intrinsically doped PEDOT do not increase substrate conductivity compared to collagen alone, enabling separate investigation into CP loading and conductivity. Interestingly, higher PEDOT loading bolsters human mesenchymal stromal (hMSC) cell gene expression of Oct‐4 and NANOG, which are key transcription factors regulating cell stemness. Conductive collagen composites with commercial PEDOT:PSS do not significantly affect the expression of these transcription factors in hMSCs. Furthermore, it is demonstrated that PEDOT regulates cellular fate independently from physical changes to the material but directly to the loading of the polymer.

## Graphical abstract

This work leverages conductive polymers with different binding and doping regimes to generate diverse conductive polymer‐collagen composites. Conductive polymer loading and bulk substrate conductivity are independently manipulated. Mechanistic cell studies show higher conductive polymer loading increases the expression of cell stemness transcription factors in mesenchymal stromal cells cultured in basal growth media.

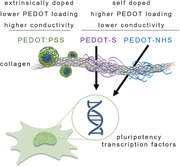

## 1 Introduction

Conductive polymers (CPs) transmit both ionic and electronic signals, show synthetic tailorability, mechanical compatibility with biological tissue, and biocompatibility, which have contributed to their prolific incorporation in regenerative biomaterials.^[^ ^1^ ^]^ It is well‐documented that this class of materials can influence cellular processes in a variety of tissue types, yet the mechanisms by which these benefits occur remain largely unelucidated. In addition to their more favorable tissue‐matching mechanics, mixed ionic/electronic conductivity has prevailed as the leading motivation for CP utilization, in many cases even serving as the benchmark for material performance. However, the direct relationship between CP properties and positive regenerative outcomes remains undetermined. Some studies have demonstrated a direct relationship between increasing conductivity and neural‐like differentiation of mesenchymal stromal cells in the presence of applied external electrical stimulation, but this trend is not ubiquitous for all cell lineages and the use of extrinsic electrical stimulation convolutes our understanding of the CP's passive role in influencing fundamental cellular processes.^[^ ^2^ ^]^ Furthermore, throughout this work, we focus on the unstimulated, or passive, properties of CP‐based biomaterials and their inherent influence on driving cellular behavior. We thus aim to better elucidate the role of specific CP‐driven materials properties on cellular phenomena.

Conductivity is only one of several interesting physical and electrochemical material properties at play when considering the incorporation of CPs into regenerative biomaterials.^[^ ^3^ ^]^ For example, composite properties such as surface charge and swelling are altered after CP incorporation, yet are not typically considered critical variables that influence physiological processes.^[^ ^4^ ^]^ Furthermore, incomplete characterization of CP composites hinders the community's ability to identify the properties most significantly influencing cell fate, and thus has limited the design of CP‐based biomaterials for regenerative applications.

In addition to the material characteristics that remain overlooked, CP‐cell interactions remain confounded by the vast number of dopant and composite blends that are frequently explored. An extensive number of CP‐dopant combinations have been synthesized with natural and synthetic host materials.^[^ ^5^ ^]^ This mixed variety of composite formulations (CP and biomaterial selection) and dopant choices, coupled with a lack of understanding for the complex chemical phenomena that enables CPs to influence cellular processes has limited their potency as biological tools. In nearly all cases, CP incorporation differentially influences cellular response, yet many studies lack a systematic explanation for the phenomena observed. Such existing studies predominantly focus on screening CP‐based constructs toward a specific tissue system, culturing cells in growth factor‐rich differentiation media with chemical cues, which distort our understanding of the fundamental cellular processes (migration, adhesion, differentiation) being directly influenced by the electroactive materials. The objective of the present study is to design and adopt a system that allows us to identify the primary variable driving the biological influence of CPs, without the confounding influences of varying dopants, base material composition, or differentiation media.

As one of the most abundant proteins in the body and a crucial facilitator of physiological ionic signals, collagen is an ideal platform to investigate (1) how CPs modify natural biomaterials they are complexed with and (2) the mechanisms by which CP composites influence cell fate. Previous studies have primarily formed CP‐collagen composites through electrochemical deposition, yet this method does not necessarily lead to homogenous substrates and can be complicated to characterize.^[^ ^6^ ^]^ We have previously demonstrated a simple approach for generating CP‐collagen composites by using a synthetic PEDOT derivative, PEDOT‐S, with the sulfonate doping group tethered to the polymer backbone. The resulting scaffolds were stable, structurally comparable to pristine collagen, and capable of deterministically influencing cell fate.^[^ ^4a^ ^]^ We have also reported another intrinsically doped PEDOT derivative, PEDOT‐NHS, which structurally resembles PEDOT‐S but covalently binds amine‐rich proteins, including collagen.^[^ ^7^ ^]^ By comparing PEDOT:PSS, PEDOT‐S, and PEDOT‐NHS‐functionalized collagen, we have developed a toolbox of diverse CP‐collagen composites with distinct electrochemical and structural properties. Despite the differences in material properties among these sponges, they share the same conjugated polymer backbone, sulfonate doping molecule, and the same base collagen material. We can thus isolate various material characteristics influenced by CP addition from the cellular processes effected.

**Figure 1:**
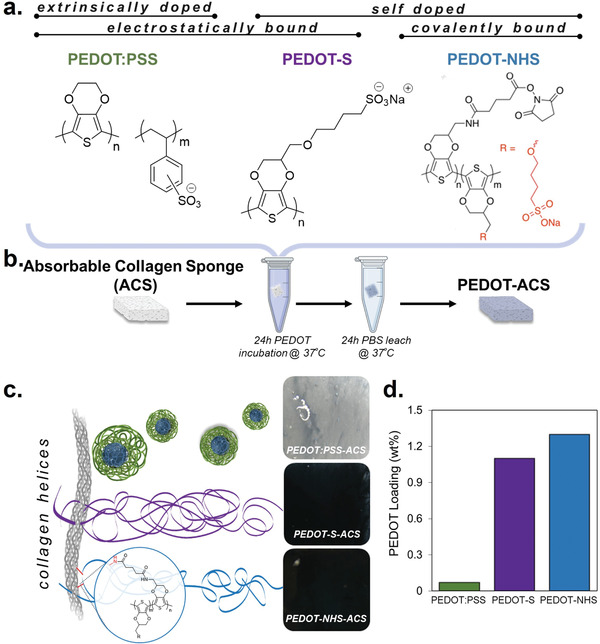
PEDOT derivatives used to functionalize collagen a) PEDOT:PSS, PEDOT‐S, and PEDOT‐NHS CPs incorporated within collagen composites. b) Functionalization procedure for PEDOT‐collagen sponges. c) Cartoon demonstrating the electrostatic binding of PEDOT:PSS and PEDOT‐S with a collagen fibril, and the covalent bonding of PEDOT‐NHS with a collagen fibril. (right) Zoomed‐in photographs of wet PEDOT‐absorbable collagen sponge (ACS) composites after incubation within each polymer for 24 h. d) XRF was performed to quantify relative sulfur within the composites, allowing PEDOT loading to be quantified.

In this study, we systematically functionalize absorbable collagen sponges (ACS) with PEDOT:PSS, PEDOT‐S, and PEDOT‐NHS, which are all different conductive/doped variants of the semiconducting polymer PEDOT (**Figure** **1**a). Material properties that have been recognized as influential in cell‐material interactions, including surface charge, modulus, and conductivity, are all examined here.^[^ ^2^, ^8^ ^]^ After analyzing the electrochemical and structural properties of the various composites, we find that PEDOT‐ACS characteristics vary depending on the PEDOT derivative and doping regime. We also demonstrate that PEDOT composites lead to elevated expression of cell stemness genes in human mesenchymal stromal cells (hMSCs), assessed through RT‐qPCR assessment of Oct‐4 and NANOG, the increase of which depends directly on CP loading and is seemingly independent of other material characteristics such as bulk conductivity and surface charge. Understanding the capability for CPs to modulate cell stemness may further elucidate the potential for conductive materials to be applied in regenerative engineering, particularly considering the seemingly widespread capabilities for CPs to induce multilineage differentiation. Cell stemness modulation has recently demonstrated remarkable potency for regenerative engineering, especially considering the increasing popularity of induced pluripotent stem cell (iPSC) applications. This is the first investigation of CP‐based materials toward influencing cell stemness, without additional external influences such as differentiation media or electrical stimulation, and thus the first demonstration of an intrinsic capability for CPs to enhance cell potency. Furthermore, we have generated a body of evidence to suggest that CPs potentiate basic cellular processes due to complex processes not limited to their ionic/electronic conduction.

## 2 Results and Discussion

### 2.1 Incubation Time Leads to Differences in PEDOT Loading in ACS

PEDOT‐collagen composites were generated by incubating absorbable collagen sponges (ACS) in either PEDOT:PSS, PEDOT‐S, or PEDOT‐NHS dispersions/solutions for 24 h (Figure 1a–c). PEDOT‐ACS was then incubated in Phosphate Buffered Saline (PBS) for 24 h at 37 °C to remove unbound polymer from the matrix. In the case of PEDOT:PSS and PEDOT‐S, complexation relies on electrostatic interactions between the negatively charged sulfonate groups and amino acids of the collagen.^[^ ^9^ ^]^ PEDOT‐NHS is unique from PEDOT:PSS and PEDOT‐S in that the NHS acts as a leaving group upon the binding of amines from collagen, enabling dual electrostatic and covalent binding regimes. Total sulfur quantification via X‐ray fluorescence (XRF) was performed to evaluate total CP loading (Figure 1d). Sulfur calibration with the known amount of sulfur per constitutional repeat unit of PEDOT‐S and PEDOT‐NHS, and the ratio of PEDOT:PSS was used to calculate the loading of PEDOT by weight in each composite. With the PEDOT‐S‐ACS and PEDOT‐NHS‐ACS, PEDOT loading was over 10‐times higher than that of PEDOT:PSS‐ACS. The differences in loading between PEDOT:PSS and intrinsically doped polymers are attributed to higher initial PEDOT concentrations of PEDOT‐S and PEDOT‐NHS, which due to their water‐soluble nature, can be dissolved at 20 mg mL^−1^, compared to stock commercially available PEDOT:PSS, in which PEDOT concentration is ≈ 2 mg mL^−1^. In addition to increased concentration, the PEDOT:PSS is a dispersion of colloidal‐like particles, which are by their nature bulkier than the dissolved intrinsically doped derivatives.

### 2.2 Composite Collagen Structure Depends on PEDOT Functionalization Scheme

**Figure 2:**
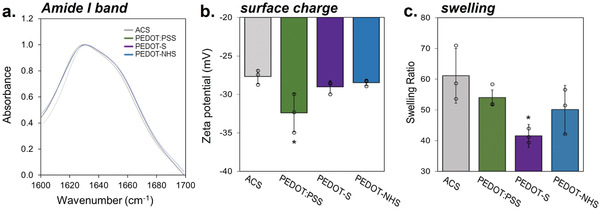
Collagen‐PEDOT interactions depend on PEDOT form a) Amide I peak of ACS and PEDOT‐ACS to screen for obvious changes in secondary collagen structure. b) Zeta potential measurements of PEDOT‐ACS composites. c) Swelling measurement of water‐collagen interactions following functionalization.

FTIR spectroscopy was utilized to understand differences in collagen‐PEDOT interaction regimes among the three different composites (Figure S1, Supporting Information).^[^ ^10^ ^]^ The Amide I band, occurring between 1600–1700 cm^−1^, is a particularly sensitive indicator of structural changes and can be used to quantify features such as secondary structure (i.e., triple helix) (**Figure** **2**a).^[^ ^1d^ ^]^ Peak shoulders of PEDOT‐ACS were broadened ≈ 1620 cm^−1^ due to the incorporation of PEDOT within the collagen matrix (Figure S2, Supporting Information). A qualitative examination of the Amide I peak revealed collagen structure remained largely unchanged across the different PEDOT‐ACS composites. The structure of natural materials, especially the triple helical arrangement of collagen, is a critical regulator of fundamental cellular behavior such as adhesion, migration, or proliferation.^[^ ^11^ ^]^ While consequences of structural distortion are widely studied in the context of metastasis, the potential for triple helical disruption to influence processes in regenerative biomaterials, and the role CPs may contribute, has been mostly unconsidered.^[^ ^12^ ^]^ Future investigations may therefore take a closer look at the fine structure of the Amide I band to deduce whether any molecular‐level features such as alpha helix or beta sheet morphologies are significantly altered with CP incorporation.

Composite zeta potential as well as swelling behavior was measured to further contextualize the influence of PEDOT on collagen (Figure 2b,c). Zeta potential demonstrates no significant difference in surface charge with the exception of PEDOT:PSS‐ACS, which is expected considering the polyanionic nature of the PSS (majority by mass) surrounding the hydrophobic PEDOT‐rich core (minority by mass). Typically the addition of CP within a biomaterial acts as an extra source of crosslinking, thus decreasing the swellability of the composite. Other CPs are also predominantly hydrophobic, which may lead to phase separation and morphological distortion due to phase incompatibilities between the hydrophilic collagen and the polymer itself.^[^ ^13^ ^]^ While all composites showed the anticipated decrease in swelling (Figure 2c), the most notable difference was observed with PEDOT‐S‐ACS. We hypothesize that decreased swelling may be attributable to high degrees of interaction between PEDOT‐S and collagen hydroxyl groups, which are key regulators of collagen‐water interactions.

**Figure 3:**
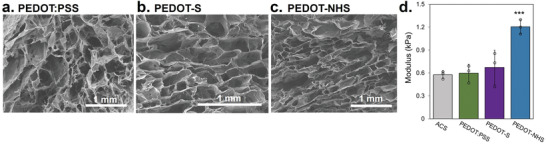
Macroscale structural properties of collagen with PEDOT incorporation a–c) SEM of macroscale scaffold topography is shown. d) The hydrated modulus of the scaffolds was calculated via compression testing.

Further analysis on the macroscale characteristics of the composites was performed, as structural and mechanical properties of substrates have been well‐documented as highly influential regulators of cell fate.^[^ ^14^ ^]^ Scanning electron microscopy (SEM) was performed to assess composite topography (**Figure** **3**a–c). Macroporous SEM images revealed no obvious pore clogging or other morphological distortions in any of the PEDOT‐functionalized composites. Porosity was quantified as previously described, and we found that there were little to no effects on the overall porous structure of the materials with PEDOT functionalization (Figure S3, Supporting Information).^[^ ^4a^ ^]^ Atomic force microscopoy (AFM) measurements corroborated that the overall topography of the materials was largely unchanged with PEDOT functionalization (Figure S4, Supporting Information). Bulk modulus was measured via compression after hydration in PBS (Figure 3d). No differences in hydrated modulus were observed between ACS and PEDOT:PSS‐ACS or PEDOT‐S‐ACS, but PEDOT‐NHS‐ACS substrates demonstrated higher moduli. Since PEDOT‐NHS covalently binds the ACS matrix, it is unsurprising that this polymer may increase the modulus while PEDOT‐S and PEDOT:PSS have no significant effect, and this result is in line with previous studies documenting the properties of EDC/NHS‐crosslinked collagen.^[^ ^15^ ^]^ Despite the statistical differences, variation in the mechanical moduli alone is not significant enough between conditions to induce changes in cellular response. Especially in the hydrated condition, all moduli are within a factor of 2, at ≈0.6–1.2 kPa.

### 2.3 Composites Demonstrate Distinct Electrochemical Behaviors Independent from PEDOT Loading

**Figure 4:**
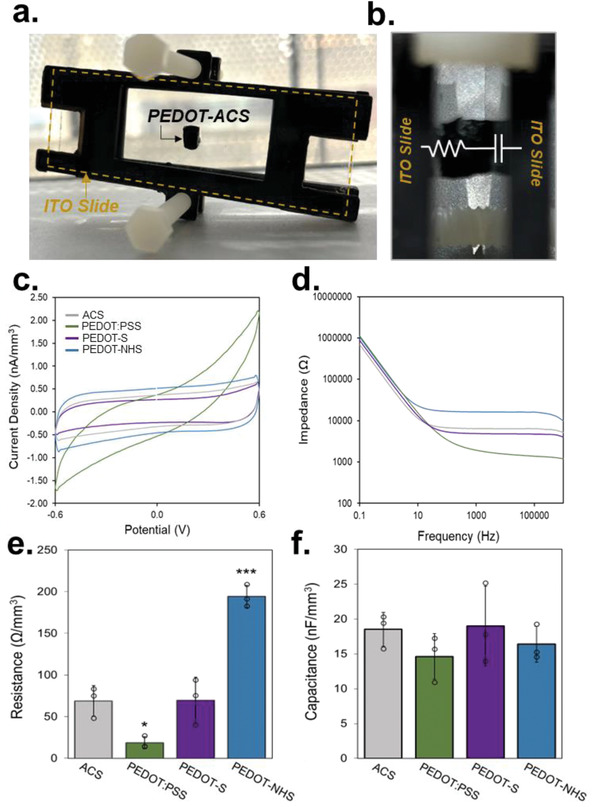
Electrochemical characterization of PEDOT‐ACS composites a,b) 3D electrochemical analysis setup is shown. c,d) CV and e,f) EIS were performed to characterize the bulk material electrochemical behavior and fit to an R‐C circuit.

Collagen has been proven as an active facilitator of physiological ionic signals in several studies, in one case even serving as electrolyte for electrochemical polymerization.^[^ ^16^ ^]^ As a critical mediator of ionic transport in vivo, any variation to collagen's native mode of ionic transport has the potential to differentially influence the target system.^[^ ^17^ ^]^ Characterizing the impedance of complex bioelectronic composites, such as those presented here, remains unstandardized. While traditional approaches such as four‐point probe offer robust methods for standard 2D films, measuring the impedance of structurally complex 3D composites is imprecise with existing 2D methods. In addition to complications associated with using four‐point probe for 3D materials, materials with low intrinsic conductivity, such as those analyzed here, are not easily assessed through this traditional dry measurement (Figure S5, Supporting Information). The construction and adaptation of a custom apparatus, however, enables electrochemical measurements that assess the bulk of the material, rather than the surface‐level conductivity. Therefore, for further electrochemical characterization, a custom setup was adopted for cyclic voltammetry (CV) and electrochemical impedance spectroscopy (EIS) measurements (**Figure** **4**a,b; Figure S6, Supporting Information). Among the CP‐based collagen composites, CV characteristics were dependent on PEDOT structure (Figure 4c). PEDOT:PSS‐ACS composites were distinct in that the current levels were nearly an order of magnitude greater than those recorded through pristine ACS. In contrast, the intrinsically doped composites, PEDOT‐S‐ACS and PEDOT‐NHS‐ACS, demonstrated cycling behavior more similar to the pristine ACS.

Electrochemical behavior was further analyzed via EIS (Figure 4d–f; Figure S7, Supporting Information). After collecting EIS spectra, the composites each demonstrated trends characteristic of an R‐C circuit. This circuit has previously been used to characterize the impedance of complex 3D biocomposites.^[^ ^18^ ^]^ None of the CP conditions used here reduced impedance, which we attribute to the low loading of the polymer within the collagen matrix. The intrinsically doped PEDOT‐NHS‐ACS and PEDOT‐S‐ACS increased resistance, whereas with PEDOT:PSS it was not impacted. Other studies have previously shown cases where CP incorporation does not reduce impedance.^[^ ^19^ ^]^ For single‐component intrinsically doped polymers such as PEDOT‐S and PEDOT‐NHS, the lack of preorganization requires prohibitively high loading to obtain bolstered conductivity. The increase in impedance with PEDOT‐NHS may be attributed to several factors including potential disruption of percolative pathways in the collagen sponge. PEDOT coatings may also increase the area of the collagen fibers, in a regime where PEDOT loading itself is not significant enough to reduce overall resistivity. Capacitance was higher with PEDOT:PSS‐ACS as compared to ACS alone, yet no other significant differences in capacitance were observed. These results emphasize the potential for dopant chemistry and CP chemical structure to alter the electrochemical properties of CP composites. Cell sensitivity to changes in substrate resistance or capacitance remains unclear, which complicates the contextualization of these electrochemical changes. While the field has predominantly focused on optimizing and analyzing bulk material conductivity, local differences in electronic properties should also be recognized as biologically potent. Here, we introduce composite systems with self‐doped CPs that have bulk electrochemical properties comparable to pristine collagen, yet the incorporation of the CP itself yields changes in the electronic microenvironment, which can still direct changes in cellular processes.

### 2.4 Self‐Doped Polymers Promote Expression of Pluripotency Transcription Factors

After physically and chemically characterizing the composites, we sought to connect the observed differences to fundamental cellular behaviors including adhesion. Topographical changes attributable to CP incorporation play a critical role in regulating cellular processes, yet these nanoscale changes are commonly neglected in characterizations of CP‐incorporated biomaterials. The interplay of topographical and electrochemical properties has been documented as highly potent for determining cell fate.^[^ ^20^ ^]^ One previous study demonstrated that neither surface topography nor applied current alone was enough to drive cells toward osteogenesis, yet with surface topography modified in tandem with an applied current, markers of late‐stage osteogenesis were observed.^[^ ^21^ ^]^ To observe cell morphology on the 3D scaffolds, F‐actin was stained 48 h after seeding human mesenchymal stromal cells (hMSCs) on PEDOT‐ACS composites (Figure S9, Supporting Information). With all composites, cells demonstrated elongated, spindle‐like morphology, which is characteristic of healthy hMSCs. Cells on ACS were anisotropic in nature, especially compared to PEDOT‐S and PEDOT‐NHS composites, with no clear directional or organizational cues from the material.

In addition to cell adhesion, the capacity for materials to direct cell differentiation is a critical metric for evaluating regenerative material efficacy, yet throughout the field, conductive materials have most prolifically been investigated in the context of a single tissue system. While several studies have demonstrated the capability for CPs to drive cells toward a specific lineage, the underlying relationship between electroactive materials and cell stemness remains poorly understood. Oct‐4 and NANOG are both transcription factors that play a key role in regulating cell differentiation, serving as key markers of cell potency.^[^ ^22^ ^]^ One previous study has shown that Oct‐4 expression can be differentially regulated with external electronic stimuli.^[^ ^23^ ^]^ Other work has demonstrated that cells transfected with pluripotency genes, including Oct‐4 and NANOG, are more effectively transduced on graphene scaffolds than tissue culture plates alone.^[^ ^23^ ^]^

**Figure 5:**
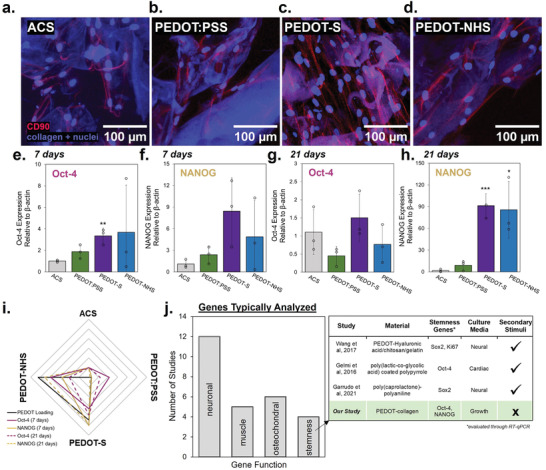
PEDOT composites distinctly affect fundamental cellular processes a–d) CD‐90 immunofluorescence was performed to verify the undifferentiated state of hMSCs 21 days after culture on PEDOT‐ACS. RT‐qPCR was also performed to measure the regulation of pluripotency transcription factors Oct‐4 and NANOG e,f) 7 and g,h) 21 days after cell seeding (n = 3). i) A radar plot is shown depicting the relationship between PEDOT loading and expression of stemness markers. j) Literature survey demonstrates widespread consideration of changes in gene expression only toward a specific tissue system in the presence of a secondary stimuli such as differentiation media or electrical stimulation, largely neglecting the overall CP‐stemness dynamics.

Cells were stained for CD90, an hMSC marker, to confirm the maintenance of hMSC phenotype on PEDOT‐ACS scaffolds throughout a 21‐day culture period (**Figure** **5**a–d). Expression of CD90 after 21 days cultured on PEDOT‐ACS shows cells did not undergo spontaneous differentiation throughout the culture duration. To further examine the passive influence of PEDOT‐based scaffolds on cell stemness, we performed RT‐qPCR to assess gene expression of Oct‐4 and NANOG after cells were cultured for 7 (Figure 5e,f) and 21 days (Figure 5g,h). With both genes assessed, cells grown on PEDOT‐S‐ACS demonstrated upregulated expression of Oct‐4 and NANOG at both 7 and 21 days. For PEDOT‐NHS‐ACS, higher expression of Oct‐4 was observed at 7 days and higher expression of NANOG was observed at 21 days compared to the pristine ACS. No significant changes in the expression of these genes were observed at any time for PEDOT:PSS‐ACS. With both intrinsically doped polymer systems, NANOG was upregulated by ≈ 100‐fold compared to the pristine ACS, pointing to a previously undocumented capability of PEDOT to not only preserve but also enhance cell stemness. Without culturing cells in a maintenance media, the inherent potential for CPs to bolster cell stemness would not be observed.

To examine whether the increase in cell stemness was conserved across cell types, human dermal fibroblasts (HDFs) were also cultured on PEDOT‐ACS. After 21 days, Oct‐4 and NANOG levels were measured to determine whether cell stemness would increase (Figure S10, Supporting Information). In contrast to the observed increase in Oct‐4 and NANOG that occurred with hMSCs, no significant changes were noted in HDFs. Although the changes in Oct‐4 and NANOG were not markedly significant compared to ACS, there was an inverse relationship between NANOG and PEDOT loading, as NANOG decreased with higher CP loadings (*p*‐value = 0.03 between PEDOT:PSS‐ACS and PEDOT‐NHS‐ACS). This data points to PEDOT influencing cell stemness in multiple cell types, although the precise mechanism by which the CP directs cell stemness is not ubiquitous and requires further investigation.

## 3 Conclusion

Throughout this study, we have assessed numerous material characteristics related to the addition of various PEDOT structures, yet only PEDOT loading directly relates to stemness marker expression (Figure 5i). While previous studies have emphasized the role of substrate conductivity in driving cellular fate, particularly with growth‐factor‐rich differentiation media, the CP itself may be uniquely altering cellular phenomena, independently from its obvious electroactivity (Figure 5j). Identification of the relationship between expression of pluripotency genes and CP loading was enabled by the use of three different CP structures. The intrinsically doped polymers specifically enabled higher PEDOT loading and thus a wider range of material characteristics were explored. More in‐depth investigation is required to elucidate the mechanism by which CPs are regulating cell stemness according to the findings here. Further pursuit of this relationship may be advanced with continued exploration of intrinsically doped CPs, rather than the conventional PEDOT:PSS, which limits overall PEDOT loading. The relationship between cell stemness and CP loading has the potential to broadly increase the capacity and impact of CPs in biomaterial applications.

## 4 Experimental Section

### Conjugated Polymer Synthesis

All manipulations of air and/or moisture‐sensitive compounds were performed under an inert atmosphere using standard glove box and Schlenk techniques. Reagents for polymer synthesis, unless otherwise specified, were purchased from Sigma–Aldrich and used without further purification. Deuterated solvents (CDCl_3_ and CD_3_OD) were purchased from Cambridge Isotopes Labs and used as received. Palladium (II) acetate was purchased from Strem Chemicals and used as received. PEDOT‐S and PEDOT‐NHS‐10 (PEDOT‐NHS) were prepared according to previously reported procedures.^[^ ^7^, ^24^ ^]^

### Biomaterial Functionalization

To functionalize the collagenous materials with PEDOT‐S and PEDOT‐NHS, a 20 mg mL^−1^ solution was prepared by dissolving the polymer in Phosphate Buffer Solution (PBS). After dissolving the polymer, 10% by volume of ammonium persulfate (1.24 m) was added to the polymer solution. For PEDOT:PSS, the solution was obtained from Heraeus and used directly from the bottle. The ACS (Integra Helistat) was submerged in the prepared polymer solution and incubated at 37 °C for 24 h. Samples were then rinsed in DI water and incubated at 37 °C in PBS overnight to ensure excess polymer diffused out of the sponge structure. Following the overnight PBS soak, the PEDOT‐ACS composites were thoroughly rinsed in DI water and lyophilized.

### PEDOT Loading Quantification

To quantify PEDOT loading, functionalized composites were dried, weighed, and analyzed via X‐ray fluorescence spectroscopy. The percent loading of PEDOT was then calculated based on the relative sulfur analysis from the integrated fluorescence of the Kα1 transition for sulfur at ≈2.3 keV of each material. The portion of the mass‐normalized integrated fluorescence from the unmodified ACS was subtracted from each composite, therefore the remaining sulfur fluorescence is from the incorporated PEDOT derivative. CP‐derived sulfur mass‐loading was calculated using a PEDOT calibration curve in unmodified collagen using PEDOT nanoparticles with one sulfur per constitutional repeating unit.^[^ ^25^ ^]^ Total PEDOT incorporation was then calculated using the known amount of sulfur within constitutional repeating units of each polymer (PEDOT‐S = 2 S, PEDOT‐NHS = 1.9 S, PEDOT = 1 S).^[^ ^26^ ^]^ As PEDOT:PSS has ≈1 wt.% PEDOT by mass, and each constitutional repeating unit of PSS (99 wt.%) has one sulfur, only ≈1.4% of the sulfur from PEDOT:PSS within the composite originates from PEDOT. XRF spectroscopy was performed in a Xenemetrix Ex‐Calibur EX‐2600 spectrometer equipped with an Rh X‐ray tube and a silicon energy‐dispersive detector. Each solid sample was placed in a plastic cup, and each collagenous material was supported on a 6 µm Mylar film. Data collection was performed under vacuum and at room temperature. The X‐ray tube was operated at 30 keV and 100 µA, and fluorescence spectra were collected for 60 s. No filter was used between the detector and sample, and L‐lines of Rh‐radiation were visible in the collected spectra. Samples were compared under the same excitation settings, and collected fluorescence was normalized by the mass of each sample.

### Zeta Potential Measurements

Zeta potential measurements were conducted by first flash‐freezing the samples in liquid nitrogen and then physically disrupting samples with a mortar and pestle. Samples were then resuspended in DI water and filtered through a 70 µm mesh. A Malvern Zetasizer was used to run the assay. Samples were evaluated according to Smoluchowski's equation. For each condition, three different samples were evaluated, and each sample was run with three different trials.

### Electrochemical Analyses

Cyclic voltammetry (CV) was performed to determine the relationship between PEDOT functionalization and ACS electrochemical characteristics. For these measurements, sponges were hydrated in DI water. CV was taken between −0.6 and 0.6 V with a scan rate of 0.1 V s^−1^ and a step size of 0.01 V for 5 scans. Additionally, electrochemical impedance spectroscopy (EIS) to evaluate differences in the frequency‐dependent response of the composites was performed. Both these techniques were performed using a PalmSens 4 and performed by securing the composites between ITO slides and secured with a sample holder as previously described.^[^ ^18^ ^]^ The ITO setup allowed for consistent spacing to be used for each experiment since screws were used to hold the materials in place throughout the duration of the test. ITO‐scaffold contact area as well as the precise spacing between samples was measured for each experiment. Resulting CVs were normalized by contact area and thickness and current density was plotted. Fits for resistance and capacitance were similarly normalized by precise scaffold size. With this approach, the samples are able to maintain their 3D structure rather than being crushed over the duration of the test, such as the case with the four‐point probe. Scaffolds were placed between the ITO such that the top of the material faced outward and electrodes were in contact with the porous portion of the material.

### Compression Testing

A 10‐mm biopsy punch was used to make consistent circular shapes for compression testing. The composite modulus was measured after incubation in PBS overnight at room temperature and compression was measured again in hydrated conditions. The tests were performed with a RSA‐G2 Solids Analyzer Dynamic Mechanical Analyzer (DMA). A linear strain rate of 2 mm min^−1^ was applied until the samples reached equilibrium. The reported modulus was calculated from the linear portion of the stress‐strain curve.

### Swelling

The swelling of the sponges was measured as previously described.^[^ ^4a^ ^]^ Briefly, the swelling behavior of PEDOT‐ACS composites was measured after soaking in PBS overnight. An initial dry weight of each individual PEDOT‐ACS was measured before and after incubation. Excess PBS was gently removed from the sponge surface by dabbing the sponge gently on a petri dish. The swelling ratio was calculated through the following Equation (1):

(1) $$swelling\;ratio\;=\;\frac{m_{f}-m_{i}}{m_{i}}*100$$

### Porosity

The overall porosity of the sponges was calculated by incubating the materials overnight in 100% ethanol.^[^ ^4a^ ^]^ Scaffolds were weighed before and after ethanol incubation. The porosity was then calculated according to the following Equation (2):

(2) $$Porosity\left(\%\right)\;=\;\frac{\left(W_{f}-W_{i}\right)\rho_{1}}{W_{f}\rho_{1}+\left(\rho_{2}-\rho_{1}\right)W_{0}}*100$$

In this equation, ρ_1_ is the density of collagen which is ≈1.21 g mL^−1^ and ρ_2_ is the density of ethanol which is ≈0.79 g mL^−1^_._

### SEM

To ensure that PEDOT functionalization did not obstruct the topography of the collagen sponge, such as by clogging the composite pores, SEM images were taken of each composite. Prior to imaging, samples were coated with ≈ 5 nm of gold‐palladium using a Denton Desk III sputter coater for macroscale images. Images were captured with a Hitachi SU8030 microscope and exported using Quartz PCI software.

### Fourier Transform Infrared Spectroscopy (FTIR)

Attenuated Total Reflection Fourier‐Transform Infrared Spectroscopy (ATR‐FTIR) was performed on a Nicolet iS50 spectrometer (Thermo Nicolet) to analyze differences in material structure following functionalization with PEDOT‐NHS. Resolution was set to 4 and 64 scans were taken. Collected spectra were baseline‐corrected using OMNIC (Thermo).

### Cell Culture and Viability

Human bone marrow mesenchymal stromal cells (hMSCs) and human dermal fibroblasts (HDFs) were acquired from ATCC and cultured according to the manufacturer's directions. Cells were cultured under 5% CO_2_ at 37 C. An alamarBlue cell viability assay was performed to screen the scaffolds for cytotoxic effects. The assay was conducted according to the manufacturer's protocol. Briefly, the test solution was diluted at 10% v/v in cell media. The samples were incubated in the solution for 4 h. Absorbance measurements were then taken and normalized to the ACS only condition. Blank samples were collected and analyzed to ensure there was no significant background attributable to signal from PEDOT.

### Confocal Microscopy

F‐actin of hMSCs was stained 48 h after cell seeding to observe cell morphology on the scaffolds. Phalloidin was used to stain the cytoskeleton as previously described.^[^ ^18^ ^]^ CD90 staining of hMSCs was performed 21 days after cell seeding on scaffolds. Fluorophore‐tagged primary antibody was obtained from Abcam (ab202512) and cells were stained according to manufacturer protocol. Cytoskeletal and CD90 images were captured using a Leica TCS SP8 Confocal Microscope and processed using ImageJ. Z‐stack images were captured with ≈ 100 µm of depth using Nikon Elements software. Image processing was then performed with ImageJ.

*RT‐qPCR* Samples from HDFs or hMSCs were lysed in TRIzol, and RNA was then harvested according to the manufacturer's instructions. RNA was diluted and stored in THE RNA Storage Solution (Thermo). RT‐qPCR was run using an iTaq Universal SYBR Green One‐Step Kit. Primer sequences are included in Supplementary Information (Table S1, Supporting Information). Samples were run with biological and technical triplicates. The gene expression was computed using the 2^−ΔΔCt^ method, normalized to *β*‐actin as the housekeeping gene.

## Conflict of Interest

The authors declare no conflict of interest.

## Supporting information

Supporting Information

## Data Availability

The data that support the findings of this study are available from the corresponding author upon reasonable request.
