# Supplementary material for: Decoupling the Influence of Poly(3,4‐Ethylenedioxythiophene)‐Collagen Composite Characteristics on Cell Stemness
Source: Adv Sci (Weinh). 2024 Feb 13;11(27):2305562. doi: 10.1002/advs.202305562 (PMC11251566; doi:10.1002/advs.202305562)
Supplement: Supplementary file 1 — Supporting Information [file ADVS-11-2305562-s001.pdf]

ADVANCED SCIENCE
Open Access

Supporting Information

for *Adv. Sci.*, DOI 10.1002/advs.202305562

Decoupling the Influence of Poly(3,4-Ethylenedioxythiophene)-Collagen Composite Characteristics on Cell Stemness

*Rebecca L. Keate, Joshua Tropp, Ruiheng Wu, Anthony J. Petty II, Guillermo A. Ameer and Jonathan Rivnay**

Supporting Information

Decoupling the influence of poly(3,4-ethylenedioxythiophene)-collagen composite characteristics on cell fate

*Rebecca L. Keate, Joshua Tropp, Ruiheng Wu, Anthony J. Petty II, Peter Kouassi, Andres, Gerena, Guillermo A. Ameer, Jonathan Rivnay**

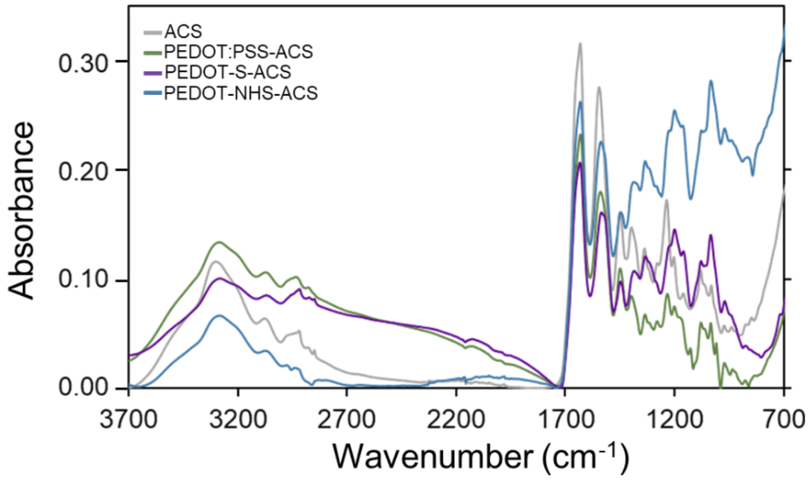

Supplementary Figure 1. FT-IR of ACS and PEDOT-ACS composites FT-IR was performed to quantify differences in molecular collagen structure following PEDOT incorporations.

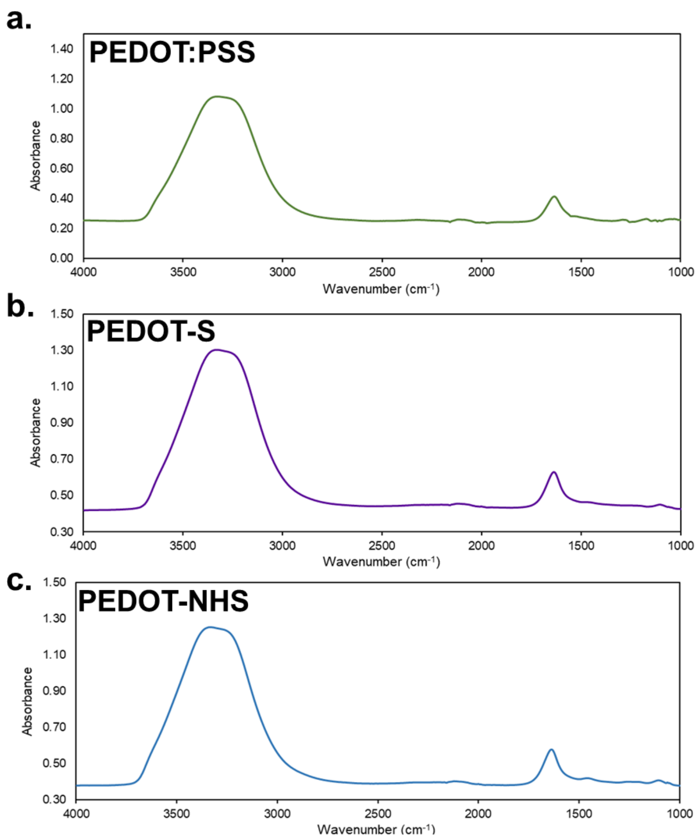

Supplementary Figure 2. FT-IR of PEDOT solutions FT-IR was performed on PEDOT solutions alone to determine the contribution of the CP specifically.

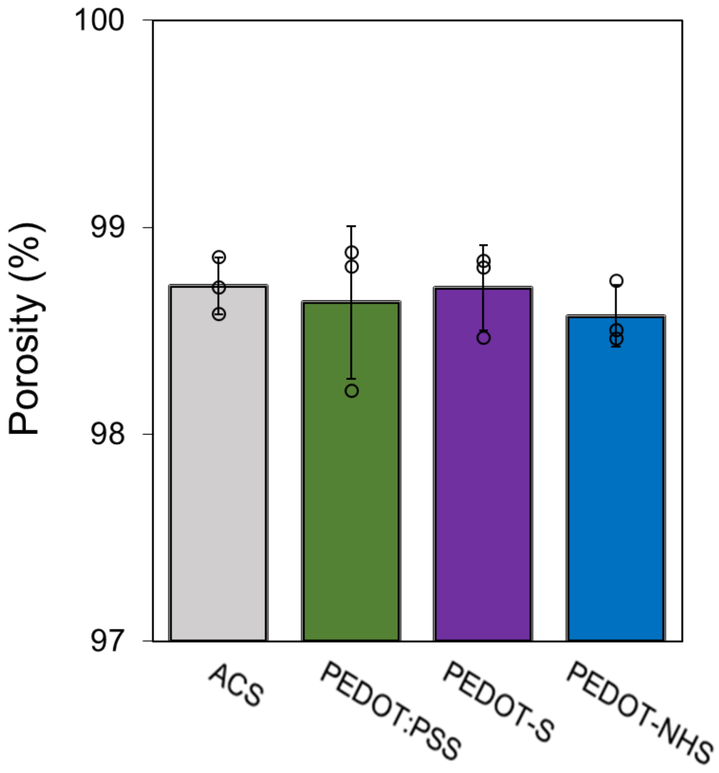

Supplementary Figure 3. Porosity measurements

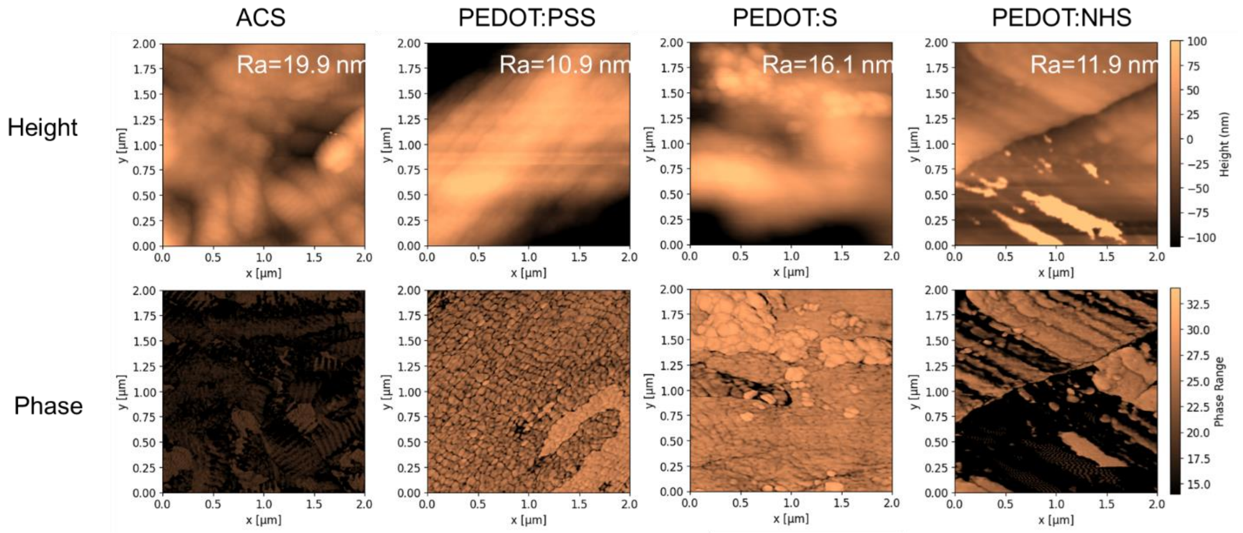

Supplementary Figure 4. Atomic force microscopy (AFM) of collagen and PEDOT-collagen sponges

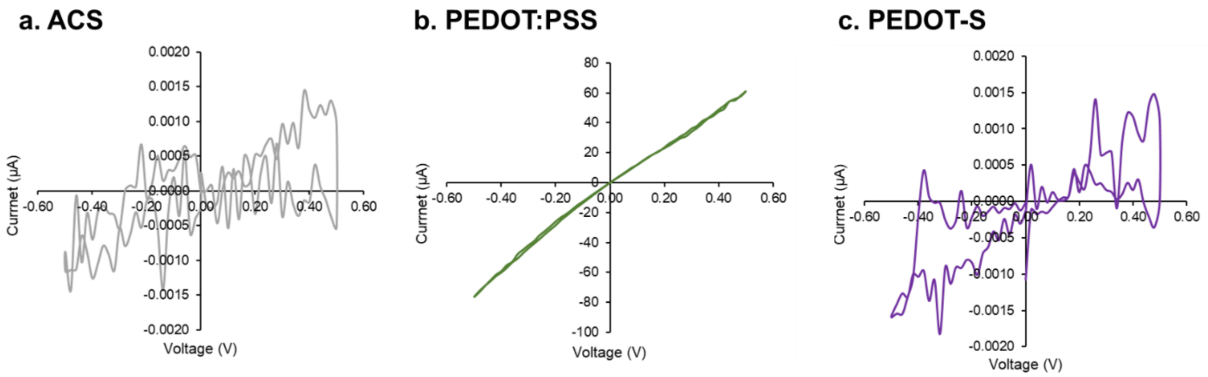

Supplementary Figure 5. Dry four-point probe measurements Representative four-point probe measurements are shown from dry (a) ACS, (b) PEDOT:PSS, and (c) PEDOT-S sponges. For materials with limited conductivity, such as ACS and PEDOT-S, the four-point probe measurements are noisy and may not be sensitive enough to fully characterize these composites.

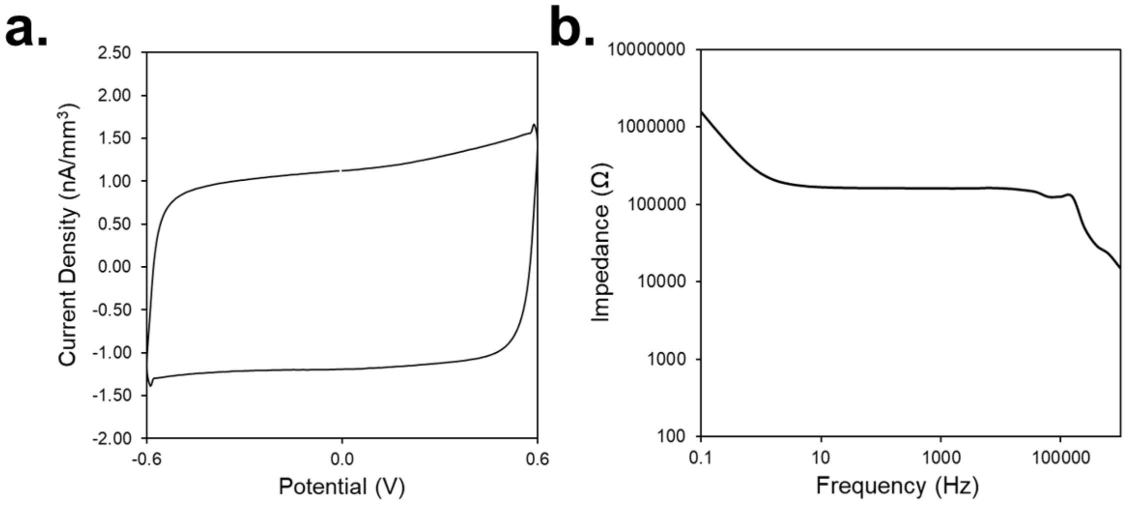

Supplementary Figure 6. DI water control for ITO setup (a) Cyclic voltammetry and (b) EIS measurements of DI water alone were performed to isolate the role of DI water from that of the scaffold.

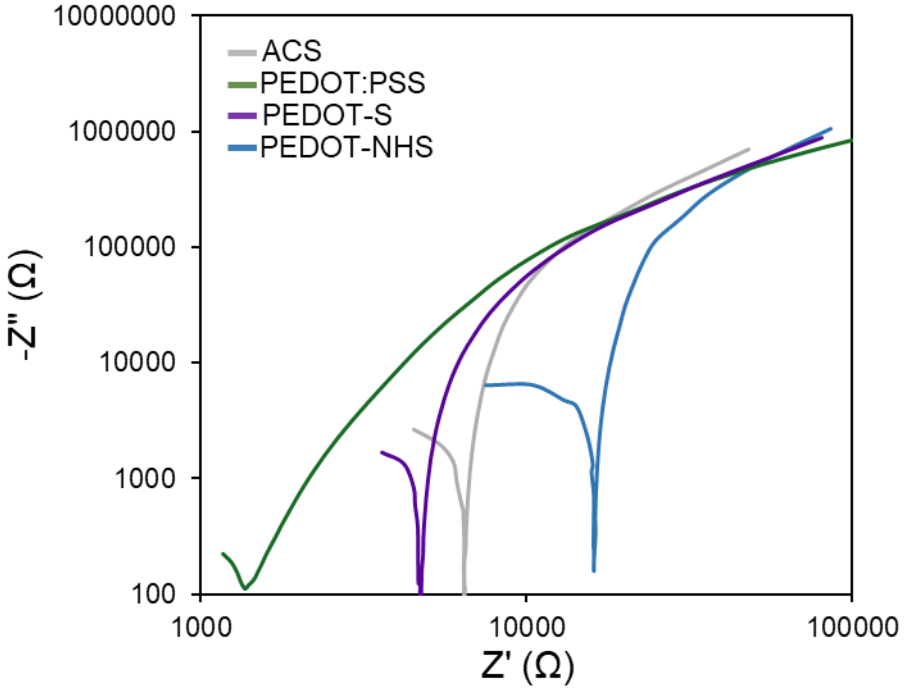

Supplementary Figure 7. Representative Nyquist plots from EIS measurements

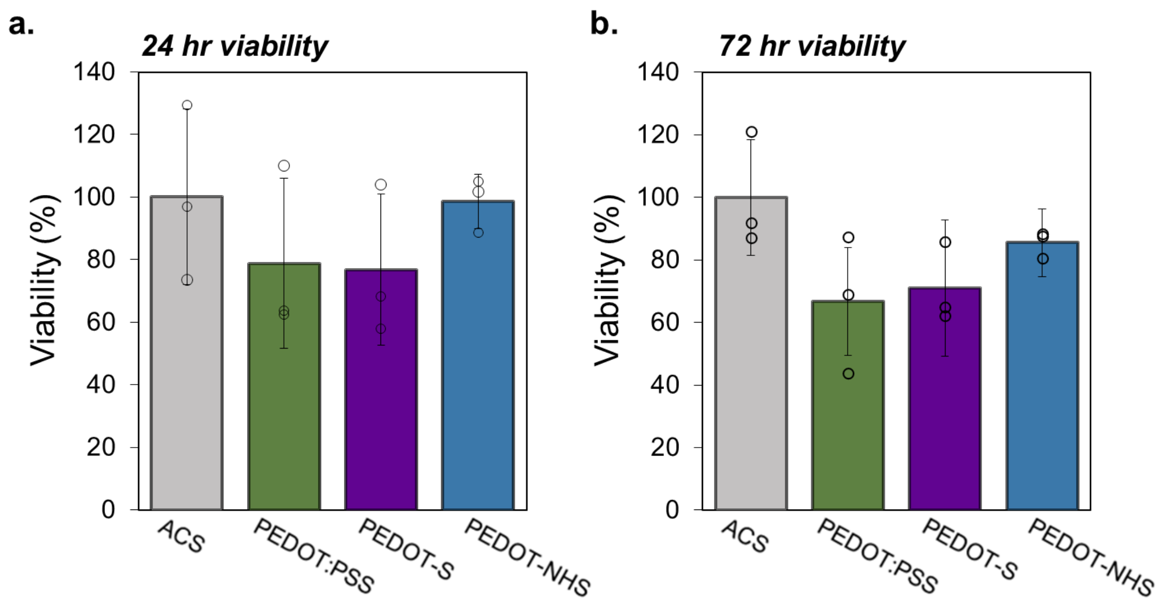

Supplementary Figure 8. PEDOT-ACS are not cytotoxic Cell viability of hMSCs (a) 24 hours and (b) 72 hours after seeding on PEDOT-ACS composites

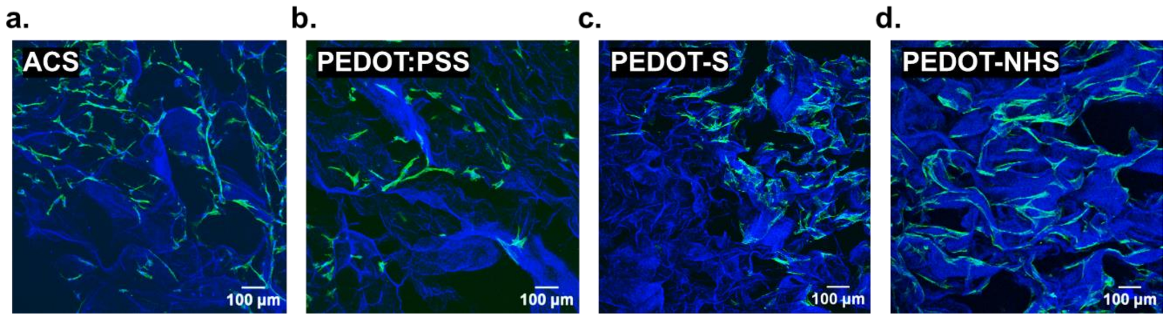

Supplementary Figure 9. Cell alignment on PEDOT-ACS depends on functionalization scheme (a-d) 3D projections of hMSCs 48 hr after seeding with phalloidin-stained actin in green and collagen autofluorescence in blue.

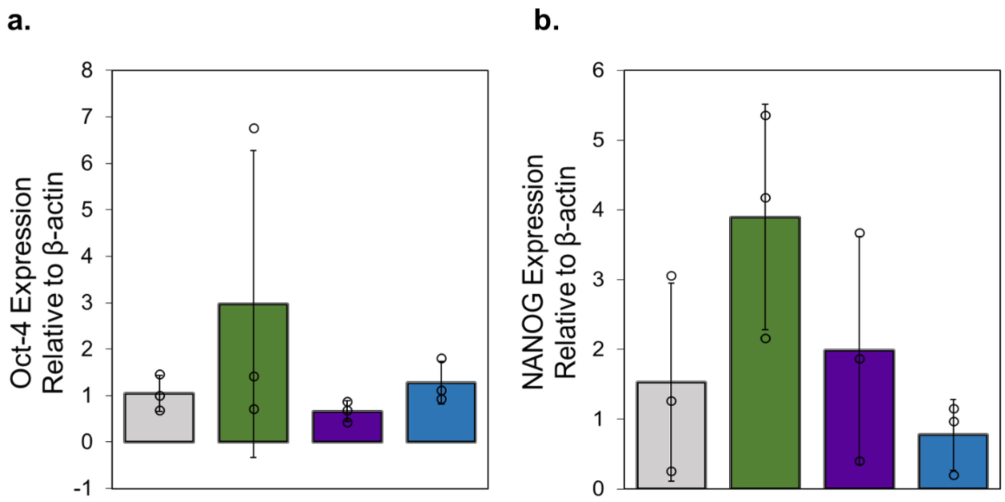

Supplementary Figure 10. Stemness of human dermal fibroblasts after 21 days assessed by (a) Oct-4 and (b) Nanog expression levels.

Supplementary Table 1. qPCR Primer Sequences

Gene	Sequence
Oct-4 Forward	GTA TTC AGC CAA ACG ACC ATC
Oct-4 Reverse	CTG GTT CGC TTT CTC TTT CG
NANOG Forward	AAT ACC TCA GCC TCC AGC AGA TG
NANOG Reverse	TGC GTC ACA CCA TTG CTA TTC TTC
β-actin Forward	ATC GTC CAC CGC AAA TGC T
β-actin Reverse	AAG CCA TGC CAA TCT CAT CTT G
